# A Minimalist Self-Localization Approach for Swarm Robots Based on Active Beacon in Indoor Environments

**DOI:** 10.3390/s23104926

**Published:** 2023-05-20

**Authors:** Mengyuan Duan, Xiaokang Lei, Zhongxing Duan, Zhicheng Zheng

**Affiliations:** 1 College of Information and Control Engineering, Xi’an University of Architecture and Technology, Xi’an 710311, China; 2 School of Marine Science and Technology, Northwestern Polytechnical University, Xi’an 710072, China

**Keywords:** swarm robots, visual localization, optical beacon, self-localization, indoor environment

## Abstract

When performing indoor tasks, miniature swarm robots are suffered from their small size, poor on-board computing power, and electromagnetic shielding of buildings, which means that some traditional localization methods, such as global positioning system (GPS), simultaneous localization and mapping (SLAM), and ultra-wideband (UWB), cannot be employed. In this paper, a minimalist indoor self-localization approach for swarm robots is proposed based on active optical beacons. A robotic navigator is introduced into a swarm of robots to provide locally localization services by actively projecting a customized optical beacon on the indoor ceiling, which contains the origin and the reference direction of localization coordinates. The swarm robots observe the optical beacon on the ceiling via a bottom-up-view monocular camera, and extract the beacon information on-board to localize their positions and headings. The uniqueness of this strategy is that it uses the flat, smooth, and well-reflective ceiling in the indoor environment as a ubiquitous plane for displaying the optical beacon; meanwhile, the bottom-up view of swarm robots is not easily blocked. Real robotic experiments are conducted to validate and analyze the localization performance of the proposed minimalist self-localization approach. The results show that our approach is feasible and effective, and can meet the needs of swarm robots to coordinate their motion. Specifically, for the stationary robots, the average position error and heading error are 2.41 cm and 1.44°; when the robots are moving, the average position error and heading error are less than 2.40 cm and 2.66°.

## 1. Introduction

Typically, swarm robots are much smaller, cheaper, and simpler than autonomous robots, and have limited sensing, computing, and loading capabilities. They often consist of dozens, hundreds, or even thousands of individual robots that work together in a coordinated manner to carry out missions. Due to their behavior being regulated based on the distributed control framework using the local sensing information, the collective behavior of swarm robots is self-organizing and emerging; it features strong robustness, scalability, flexibility, and adaptability [1]. These excellent characteristics enable them to serve a wide range of challenging applications, such as target search [2], collective transport [3], multi-target trapping [4], object collection [5], etc. Recently, the field of swarm robotics has attracted a lot of attention, from theoretical approaches to various applications.

For many tasks within indoor environments, such as environmental maintenance, structure construction, search and rescues, etc., swarm robots can provide unique solutions and have efficiency advantages due to the fact that they can act in parallel and work cooperatively. The corresponding swarm-robotized solutions for these tasks in literature are known as collective waste collection [6], collective robotic construction [7], collective search and rescues [8], etc. Of particular interest is when robots carrying specialized equipment have to enter structurally damaged or even collapsed buildings to undertake searches and rescues of survivors, as is the situation when dealing with disaster scenarios such as earthquakes, typhoons, and fires. Swarm robots can take full advantage of them due to their small size, fast deployment, and large-scale parallel activities to perform tasks such as casualty search, goods transport, and structural repair of buildings. However, it remains a challenge for swarm robots to effectively locate themselves when working inside buildings. We emphasize that effective self-localization is a necessary condition for swarm robots to successfully fulfill these indoor tasks.

The commonly used robot localization methods such as GPS, UWB, and SLAM, are not suitable or cannot meet the requirements of swarm robots working indoors. Some of the restrictive reasons are as follows: Firstly, the electromagnetic signal might be blocked by the exterior wall and roof of buildings, making GPS [9] unstable or even completely ineffective indoors. Secondly, other localization methods such as UWB, motion capture systems, and wireless sensor location [10,11,12] require the prior installation and calibration of the external auxiliary systems, which makes them inflexible in using, and impossible to deploy quickly. Thus, these methods are not suitable for the indoor localization of swarm robots in emergencies. Finally, the localization methods based on laser and/or visual SLAM [13,14] are also challenging to implement in swarm robots due to the restriction of small size, limited power supply, and poor on-board computing.

As a consequence, a minimalist and effective self-localization approach with low computational costs and rapid deployment is required for swarm robots working indoors. It is the goal of our study in this paper.

### 1.1. Related Works

Self-localization in an indoor environment is one of the challenging issues for swarm robotics. Most current relevant efforts are based on infrared (IR) sensors. Typically, IR sensors are used to collect relative bearing and distance data between individual robots, then this positional data is then aggregated and integrated by specific methods to obtain information about the spatial configuration of the robot swarm. For example, Mao et al. [15] used both a compass and an IR transceiver to achieve relative positioning of swarm robots, where the former establishes a common reference direction for all robots and the latter detects the relative positions of neighboring robots. Kim et al. [16] proposed a method for estimating the topology of swarm robots, in which six pairs of IR transceivers are used by each robot to obtain the relative positions of its neighbors; this information is integrated by a central computer to estimate the swarm’s topology and generate a map to characterize the distribution of swarm robots. Wang et al. [17] proposed a relative localization method for swarm robots based on the so-called polar method, which similarly employs IR sensors for relative sensing between robots. Nevertheless, we must be aware that the angular resolution and radial distance resolution of these IR-based localization methods are very limited due to the sensing nature and layout of IR transceivers.

The shortcomings in angular and radial distance resolution of the above IR-based localization methods can be compensated by using high-resolution visual sensors in swarm robots [18]. For instance, Bonani et al. [19] designed a novel omnidirectional camera for MarXbots swarm robots, which consists of a monocular visual sensor and a uniquely designed convex reflector. This visual module allows each MarXbots to obtain the position information of all its surrounding neighbors with high resolution. Wang et al. [20] proposed a visual perception method for swarm robots by a using forward-facing visual sensor, in which swarm robots determine the relative position, orientation, and velocity of their neighboring robots by observing the optical beacon mounted on top of each robot. It is worth noting that the aforementioned positioning methods, whether based on infrared or visual sensors, suffer from two shortcomings: achieving group-level configuration of swarm robots requires the exchange of relative positioning information between individuals, and this process leads to a significant increase in communication load that grows exponentially with the number of robots, known as “communication congestion [21]”. Furthermore, when these methods are used in large and dense swarms, both infrared and visual sensors may become invalid due to mutual occlusion between robots.

When a robot works indoors, the most favorable condition for navigation and localization is the structured building. The ceiling, like the floor, is a building-scale architectural reference plane that is continuous without gaps and has a flat surface and good reflectivity. In particular, the ceiling is generally parallel to the floor and the view observed from the ground is not easily obstructed by obstacles. By projecting or laying customized visual beacons on the ceiling, the robot can achieve self-localization. For instance, Li et al. [22] pre-placed a series of artificial markers on the ceiling that encoded each marker’s ID and some positional reference information, and the mobile robot locate its position autonomously by observing these markers. 

### 1.2. Motivation and Contributions

In nature, animal domains have developed efficient division of labor mechanisms such as a head goose leading young geese to migrate long distances [23], and a scout bee broadcasting the location information of nectar source to other bees with a tail-wagging dance [24]. Inspired by this simple and efficient collaborative idea, we propose an active-optical-beacon-based cooperative localization approach for indoor working swarm robots in this paper. Specifically, a robotic navigator is introduced into a swarm of robots to project artificial beacons on the ceiling. Each individual in the robot swarm locates itself relative to the navigator by monitoring the artificial beacon through a monocular camera with a bottom-up view mounted on the top of the robot’s body. Basically speaking, the method can provide a limited range of location services to swarm robots when the navigator’s position remains stationary; however, the range of location services can be extended by shifting the navigator’s position. It should be noted that despite being a minimalist approach to localization, this method proves to be efficient and rapidly deployable for swarm robots with limited computing capabilities. This is due to the fact that there is no requirement for any external auxiliary equipment to be pre-installed at the work site.

In this paper, we focus on stationary navigator and concern on the following issues: ceiling-beacon-based localization approach of swarm robots, designing and recognizing method of optical beacon, and analyzing the self-localization performance of our method in different scenarios. The main contributions of this paper are as follows:

(1) A cooperative localization approach for indoor swarm robots is proposed, in which a robotic navigator is introduced to project an optical beacon on the building’s ceiling, and swarm robots locate their positions by observing the beacon. The advantages of this approach are considered to be minimalist, and efficient, and with no requirement for auxiliary equipment.
(2) Our unique ceiling-projected beacon and bottom-up visual observation have two main advantages. On the one hand, relative localization failures caused by mutual visual occlusion can be successfully avoided. On the other hand, recognition complexity caused by dynamic environments such as people’s movement and unstructured furniture can be eliminated. In this sense, our approach is suitable for large and dense groups of swarm robots working indoors.
(3) The proposed approach is verified through self-localization experiments using the real robot, and its localization precision is sufficient for the cooperative operation of swarm robots.

### 1.3. Structure of the Article

The remainder of this paper is organized as follows. In Section 2, a detailed description of the proposed localization approach for swarm robots is provided. In Section 3, we explain the implementation of our self-localization approach in detail. Real robotic experiments are presented to verify the effectiveness of our approach in Section 4. Finally, in Section 5, we conclude this paper by summarizing our results and discussing some potential avenues for future work.

## 2. Localization Approach for Swarm Robots

When swarm robots work indoors, the ceiling is an ideal beacon display plane due to its flat surface, good reflectivity, and continuity without gaps. If we project an active optical beacon onto the ceiling, by observing the optical beacon, the robot can determine its relative position with respect to the coordinate system defined by the beacon. The strategy of bottom-up observation reduces the visual recognition complexity brought by dynamic environments and avoids visual occlusion among swarm robots. With these considerations in mind, we propose a self-localization methodology for indoor working swarm robots based on actively projecting an optical beacon on the ceiling.

**Figure 1:**
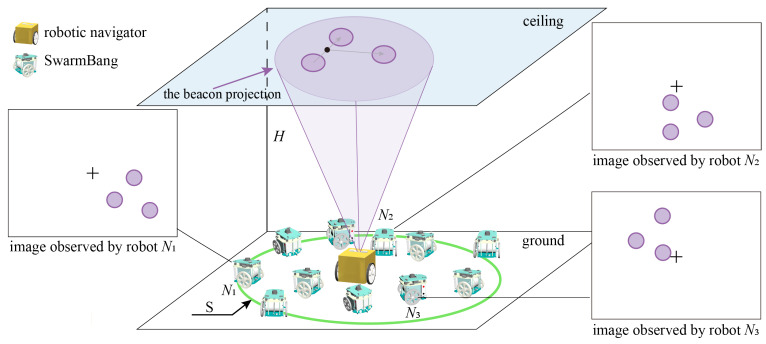
Schematic diagram of the proposed self-localization approach for swarm robots.

Assuming that the ceiling is flat with good reflectivity, within a swarm of robots, an autonomous mobile robot (referred to as *Navigator*) is introduced into a group of swarm robots to provide localization services. As shown in Figure 1, the robotic navigator offers several capabilities such as image projection, autonomous navigation, remote-operated motion control, global positioning, wireless communication, and more. Its most unique feature is the ability to project a variable size, adjustable brightness, and colored optical beacon onto the ceiling. This optical beacon must be visible and easily identifiable and contains the local coordinate system information required for determining the robot’s position and orientation, such as the common reference direction and coordinate origin.

Our SwarmBang miniature swarm robot is designed to perform specific indoor tasks (see Section 3 for details). Contrary to the versatile robotic navigator, the SwarmBang robot is minimalist designed and highly task-oriented. Each swarm robot is equipped with a visual module mounted on top to observe the optical beacons projected onto the ceiling. By measuring the positional discrepancy of the optical beacon from the robot’s view, SwarmBang is able to determine its relative position and orientation with respect to the optical coordinate system (defined by the optical beacon). Subsequently, by integrating and processing the relative localization information of each individual robot, the robotic navigator can identify and establish the global coordinates, spatial distribution, and collective movement status of the entire swarm. On the one hand, this group-level information can be used by swarm robots for task planning and decision-making. On the other hand, this swarm-robotic information can also be communicated to a remote control center so that the human operator can monitor and/or intervene in the operations of swarm robots.

Due to the limited viewing range of the visual positioning module, a beacon, which is fixed at a specific location, can only provide location services for SwarmBang robots in a limited region. This service region is determined by the size and shape of the beacon, the view field of the robot’s positioning module, and the height of the ceiling. In order to provide a wide range of localization services for swarm robots, once the swarm robots have completed their tasks in a certain area, the robotic navigator moves to the next location and projects the optical beacon again according to the task requirements of the swarm robots in the next stage. As mentioned above, one of the capabilities of the robotic navigator is to obtain its global position. Thus, the robotic navigator can translate the group status and position of the swarm robots in the beacon coordinate system to the global coordinate system.

In this study, we concentrate on implementing the self-localization of swarm robots, specifically how they determine their relative position and heading based on the active optical beacon on the ceiling, and analyzing the self-localization performance of our proposed approach in different scenarios. We primarily focus on two major issues: the design and recognition of active beacons, the relative localization approach of swarm robots based on active beacons.

## 3. Implementation of Self-Localization Approach

In this section, the implementation of our self-localization approach is provided in detail, including the introduction of our swarm robot and its visual localization module, the design and recognition method of active beacons, and the relative localization method based on the active beacon.

### 3.1. Miniature Swarm Robots

**Figure 2:**
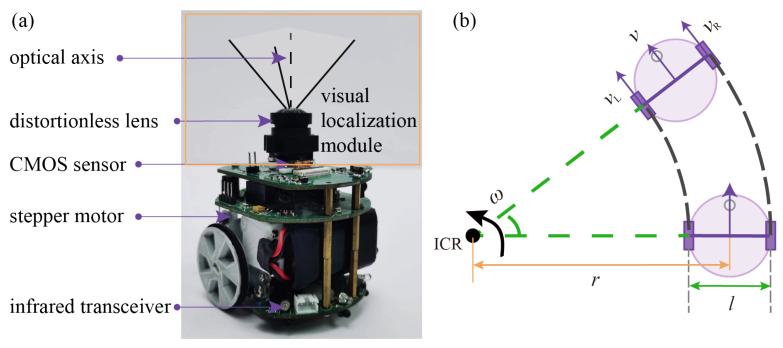
The SwarmBang robot and its kinematics model: (**a**) SwarmBang with a visual localization module; (**b**) kinematics model.

Our swarm robot is named SwarmBang, and its hardware architecture is shown in Figure 2a. The SwarmBang robot is a miniature mobile robot, weighing 180 g, 60 mm in diameter, and 80 mm in height. The main hardware components of the SwarmBang robot include the low-power microcontroller responsible for central computation, two 5 V stepper motors driving the robot’s wheels, an omnidirectional wheel to maintain balance, and three pairs of infrared transceivers for local emergency avoidance. Our SwarmBang robot can be equipped with a variety of functional components, including brushes, gas sensors, grippers, and others, to perform various indoor tasks, such as waste cleaning, fire source detection, and collective object transport.

The visual localization module of the SwarmBang robot is a miniature monocular camera that is developed based on the open-source platform, OpenMV4. The module is mounted horizontally on top of the robot, and the camera’s field of view is oriented vertically upward to ensure that the imaging plane remains parallel to the robot’s motion plane. The localization module has external dimensions of 60 × 43 × 18 mm and is mounted approximately 70 mm above the ground. In terms of hardware configuration, the module includes a low-power STM32H7 processor with 480 MHz main frequency, 1 MB RAM, and 2 MB Flash memory, a CMOS sensor with 5-megapixel resolution (2592 pixels × 1944 pixels), and a distortion-free lens with 2.8 mm focal length and F2.5 aperture. Using this module, the SwarmBang robot can observe the optical beacons on the ceiling and determine its relative position.

As shown in Figure 2b, the SwarmBang robot adopts a two-wheel differential motion structure. Instead of directly controlling the speed of the left and right wheels, SwarmBang’s motion is regulated by setting its linear and angular speed. Its control command is updated at a fixed time interval Δt. During each update cycle, the robot maintains its linear and angular speed until a new control command is received. A brief overview of the control process is provided as follows: At any moment, the robot’s movement can be seen as a rotation around point ICR. During this rotation process, the robot’s angular speed ω is equal to the angular speeds $\omega_{L}$ and $\omega_{R}$ of its left and right wheels. Therefore, the linear speeds $v_{L}$ and $v_{R}$ are related as follows:

(1) $$v_{L}(r+\frac{l}{2})=v_{R}(r-\frac{l}{2})$$

where *r* is the turning radius of the robot, and *l* = 48 mm is the distance between the two wheels. Using the relationship between the angular speed ω, turning radius *r*, and linear speed *v*, we can obtain:

(2) $$\omega =\frac{v_{R}}{r+\frac{l}{2}}=\frac{v_{R}-v_{L}}{l}$$

(3) $$v=\omega \times r=\frac{v_{R}-v_{L}}{l}\times \frac{l\left(v_{R}+v_{L}\right)}{2\left(v_{R}-v_{L}\right)}=\frac{v_{R}+v_{L}}{2}$$

When the desired linear and angular speeds of the robot are known, the speeds of the left and right wheels can then be used to drive the robot’s movement.

(4) $$\begin{array}{l} v_{L}=v-\frac{\omega l}{2}\\ v_{R}=v+\frac{\omega l}{2} \end{array}$$

For the SwarmBang robot, its maximum linear speed $v_{\max}=120\mathrm{mm}/s$, and its maximum angular speed $\omega_{\max}=86\deg/s$.

### 3.2. Design and Recognition of Optical Beacons

The optical beacons should include information such as the origin and a directional reference to help the robot perform self-localization easily and accurately. Also, it must be extracted and recognized quickly and accurately. In this section, we present a novel scheme for designing optical beacons and provide a fast recognition and verification method.

#### 3.2.1. Design of Optical Beacons

**Figure 3:**
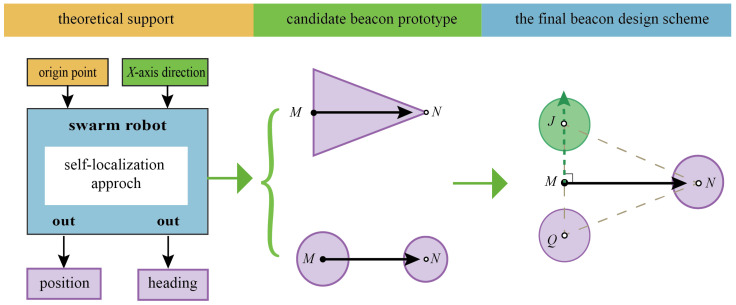
Conceptional design of the active optical beacon.

As mentioned in the previous section, active optical beacons should provide robots with information about the origin and reference direction of the coordinate system. Considering this requirement, two candidate beacon prototypes are shown in Figure 3. The first prototype beacon is designed as an isosceles triangle, while the second prototype beacon is designed as two circles with different diameters. For the former, the midpoint of the triangle’s base is defined as the origin point *M* of the beacon coordinate system; the vertex of the triangle that is not on the base is point *N*, which is a point on the *X*-axis of the beacon coordinate system. For the latter, the center of the big circle is the origin point *M* of the beacon coordinate system, and the center of the small circle is point *N*. For them, the direction of the *X*-axis points from *M* to *N*.

Although the above-mentioned prototype designs are theoretically feasible, it is challenging for swarm robots to ensure the stability of recognition when they actually detect and recognize these beacons. This is due to the relatively high computational complexity of the first prototype. The second prototype has a regular shape that can be recognized effortlessly. However, it is easy to reverse the direction when determining the positive *X*-axis direction by comparing the sizes of the two circles. 

Here, we choose to integrate the two prototype designs, and the final beacon design scheme (referred to as triangular isomorphic beacon) is shown in Figure 3. The triangular isomorphic beacon is composed of three equally sized circles, arranged in an isosceles triangle. On the basis of identifying the centers of the three circles and measuring the distance between each pair of circles, the base and sides of the triangular beacon can be determined. As shown in Figure 3, the beacon coordinate system can then be established: the origin point is the midpoint, *M,* of the triangular base; the positive direction of the *X*-axis directs from point *M* to point *N* (referred to as the vector $\vec{MN}$), where *N* is the vertex that is not on the triangular base; the direction from point *Q* to point *J* is the positive direction of the *Y*-axis (referred to as the vector $\vec{QJ}$).

It is worth noting that due to the inherent distortion of optical lenses, some errors in beacon recognition inevitably occur at the edge of the camera’s field of view. Therefore, the visual beacons should contain specific verification information so that the robot can quickly check whether to accept or discard the current recognition result. Our designed triangular beacon supports geometric verification based on the property of isosceles triangles, i.e., the midline and base are perpendicular to each other. Specifically, if the vector $\vec{MN}$ is not perpendicular to the vector $\vec{QJ}$, the beacon recognition result should be discarded.

#### 3.2.2. Recognition and Verification of Beacons

**Figure 4:**
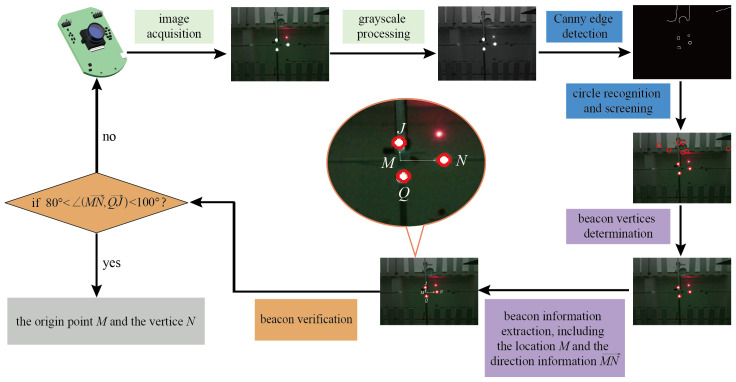
Flowchart of active optical beacon recognition and verification.

In order to recognize and extract the structural information of the triangular isomorphic beacon we designed, we propose a recognition processing flow for the visual localization module, as shown in Figure 4. The flow primarily consists of three segments: beacon detection, extraction of localization information, and information verification.

Firstly, the color images collected by the robot are converted to grayscale. By using Canny edge detection [25] and Hough circle recognition [26], the coordinates of possible vertices in the robot’s field of view are acquired. Here, spots with a radius of less than three pixels are considered as noise and are removed.

Secondly, we scan and mark all the candidate vertices based on the NCC template matching approach [27] to determine the three vertices of the optical beacon, i.e., the centers of the three circles. On the basis of calculating the distance between each pair of vertices, the base, and waist of the beacon can be determined by vertex-spacing sorting, and the pixel coordinates of three vertices *N*, *J*, and *Q* are extracted. Then, according to the design scheme described in Section 3.2.1, we can obtain the information of the coordinate system as: the origin point is M=(J+Q)/2, and the positive directions of the *X*-axis and *Y*-axis are the vector $\vec{MN}$ and $\vec{QJ}$, respectively.

Finally, the detected beacons are verified based on the geometric property of the isosceles triangle, i.e., its midline and base are perpendicular to each other. Formally, the inner product of the vectors $\vec{MN}$ and $\vec{QJ}$ should be zero if they are perpendicular. However, the actual recognition process of the beacon may be impacted by unfavorable factors such as camera distortion, stray light interference, and blurred contours, which may lead the robot to incorrectly identify the three vertices. Therefore, a criterion is set as follows: if the angle between the vectors of $\vec{MN}$ and $\vec{QJ}$ is in the range of $\left(80^{\circ },100^{\circ }\right)$, the recognition result is valid for the current frame; otherwise, we consider that the recognition result is improper and the robot re-collects a new frame of the image to recognize and extract the efficient data.

### 3.3. Relative Localization

When the swarm robots perform certain tasks in an indoor area, the robotic navigator remains stationary in a fixed position and projects optical beacons on the ceiling. Along with the movement of the robot, the imaging of the beacon within its field of view changes with the change of its pose. Utilizing the imaging difference and prior knowledge of the beacon’s pixel coordinates, the position and heading of the robot relative to the beacon coordinate system can be estimated.

**Figure 5:**
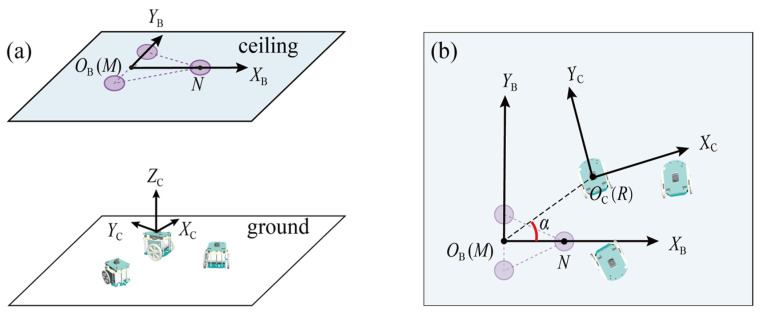
Robot’s position and heading calculation: (**a**) 3D scenario; (**b**) top view of beacon coordinate system.

As shown in Figure 5a, two coordinate systems are established: (1) the beacon coordinate system $X_{B}O_{B}Y_{B}$, which serves as the reference coordinate system for the self-localization of swarm robots; (2) the camera coordinate system $X_{C}Y_{C}Z_{C}$, in which the origin point is the optical center of the robot’s camera (which is coincident with the robot’s motion center), and the positive directions of the *Y*-axis and *Z*-axis are aligned with the robot’s heading and the camera’s optical axis, respectively. For a certain frame of the image, the robot first determines the coordinates of the beacon in the pixel coordinate system, MP and NP. Then, MP and NP are converted into the corresponding coordinates MC and NC in the camera coordinate system. Finally, according to the geometric relationship between $X_{C}Y_{C}Z_{C}$ and $X_{B}O_{B}Y_{B}$, the position and heading of the robot in the coordinate $X_{B}O_{B}Y_{B}$ are obtained.

According to the camera pinhole camera model [28], the relationship between a point’s position in the camera coordinate system and its position in the pixel coordinate system is as follows:

(5) $$Z_{C}\left[\begin{array}{l} u\\ v\\ 1 \end{array}\right]=T\left[\begin{array}{l} X_{C}\\ Y_{C}\\ Z_{C} \end{array}\right],\;T=\left[\begin{array}{ccc} \frac{1}{dx} & 0 & u_{0}\\ 0 & \frac{1}{dy} & v_{0}\\ 0 & 0 & 1 \end{array}\right]\left[\begin{array}{ccc} f & 0 & 0\\ 0 & f & 0\\ 0 & 0 & 1 \end{array}\right]$$

where $Z_{C}$ is the distance between the imaging object’s plane and the robot’s camera optical center, (u,v) denotes the coordinates in the pixel coordinate system, and $\left(X_{C},Y_{C},Z_{C}\right)$ denotes a point in the camera coordinate system. T is a 3 × 3 matrix of the camera’s internal parameters, which can be obtained through several camera calibration methods [29]. *f* is the focal length of the camera lens. $\left(u_{0},v_{0}\right)$ is the pixel coordinates of the camera’s optical center. *dx* and *dy* represent the physical dimensions of a unit pixel in the *X*-axis and *Y*-axis, respectively.

For an indoor working robot, due to the ground and ceiling being parallel, $Z_{C}$ is constantly equal to *h*, i.e., $Z_{C}=h$, if we ignore the impact of ground undulation on the robot’s motion, where *h* is the distance between the ceiling plane and the optical center of the camera. Hence, Equation (5) can be re-written as:

(6) $$h\left[\begin{array}{c} u_{k}\\ v_{k}\\ 1 \end{array}\right]=T\left[\begin{array}{c} x_{k}\\ y_{k}\\ h \end{array}\right],\;T=\left[\begin{array}{ccc} a_{x} & 0 & u_{0}\\ 0 & a_{y} & v_{0}\\ 0 & 0 & 1 \end{array}\right]$$

where $\left(u_{k},v_{k}\right)$ and $\left(x_{k},y_{k},h\right)$ stand for any point K’s coordinates in the beacon coordinate system and camera coordinate system, respectively. $a_{x}=f/dx$ and $a_{y}=f/dy$, respectively, are the scale factors of the horizontal and vertical axes for the image coordinate system. Then, based on the inverse transformation of Equation (6), the point’s positions, $\left(u_{k},v_{k}\right)$, in the camera coordinate system can be obtained:

(7) $$\left[\begin{array}{c} x_{k}\\ y_{k}\\ h \end{array}\right]=hT^{-1}\left[\begin{array}{c} u_{k}\\ v_{k}\\ 1 \end{array}\right]$$

Based on Equation (7), the pixel positions MP(um,vm) and NP(un,vn) acquired by the visual localization module can be translated into the camera coordinate system, yielding MC(xm,ym,h) and NC(xn,yn,h), respectively. Because the optical axis of the visual localization module is perpendicular to the ceiling plane and the motion center of the robot coincides with the origin of the camera coordinate system, the robot’s motion can be mapped to the ceiling plane on which the beacon is projected. Therefore, as shown in Figure 5b, according to the geometric relationship between the coordinate systems $X_{B}O_{B}Y_{B}$ and $X_{C}Y_{C}Z_{C}$, the position of the robot in the beacon coordinate system, $\left(x_{r},y_{r}\right)$, is as following:

(8) xryr = MC−RCcosαsinαα =arccosMCRC→MCRC→⋅MCNC→MCNC→

where RC is the origin of the camera coordinate system, i.e., RC=(0,0); ’⋅’ represents the inner product of the vectors. 

Finally, since the robot’s heading that we set is concordant with the positive direction of the *X*-axis of the camera coordinate system, its heading is equal to the rotation angle of the camera coordinate system with respect to the beacon coordinate system:

(9) $$heading\;\;=\arctan2(y_{n}-y_{m},x_{n}-x_{m})\;\;$$

where arctan2(*,*) stands for the four-quadrant arctangent function.

## 4. Experimental Results

In this section, some real robot experiments are conducted to test the localization performance of our approach. We first carry out an experiment to investigate the effective localization area of the robotic navigator, and then, two experiments with stationary and moving robots, respectively, are performed to verify the feasibility and effectiveness of swarm robots.

### 4.1. Experiment Set-Up 

**Figure 6:**
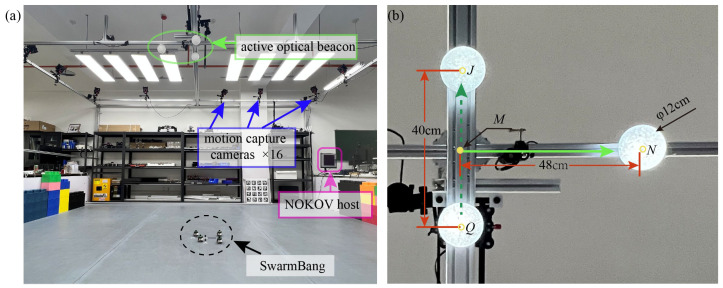
Experimental set-up for self-localization of swarm robot: (**a**) Experimental arena; (**b**) active optical beacon.

As depicted in Figure 6a, our experiments are conducted in a ~30 m^2^ indoor arena with flat ground and no obstacles. Around the arena, 16 NOKOV motion capture cameras are mounted, which connect to a host. This motion capture system serves to monitor the real-time position and heading of each SwarmBang robot. The NOKOV system has a positioning and orientation accuracy of 0.2 mm and 0.28°, respectively, so these collected data can be treated as the true pose to check the robot’s self-localization results.

In the original setup of our approach, an optical beacon should be projected onto the ceiling by the robotic navigator. For the purpose of simplifying the experimental setup, in practical experiments, three fixed light spheres are used as the beacons. This setting also helps to minimize the interference in localization performance caused by a cluttered background, changes in the projection position, and the jitter of the projected pattern. Specifically, three white lights are set up at the top of the site to simulate the active beacons projected by a robotic navigator on the ceiling. Figure 6b illustrates the location and spacing of three lights, that are installed at a height of 2.7 m above the floor.

To quantitatively evaluate the self-localization performance of SwarmBang robots, the following metrics are used.

(1) The position error, $E_{\mathrm{pos}}$, is defined as the Euclidean distance between the true position and the estimated position of the self-localization module:

(10) $$E_{\mathrm{pos}}=\left\|\left(x_{s},y_{s})-(x_{a},y_{a}\right)\right\|$$

where $\left(x_{s},y_{s}\right)$ and $\left(x_{a},y_{a}\right)$ are the self-localized coordinates and the true coordinates, respectively. The positioning accuracy of the robot decreases as $E_{\mathrm{pos}}$ increases. $E_{\mathrm{pos}}=0$ is the ideal case.

(2) The heading error, $E_{\mathrm{ang}}$, is defined as the absolute value of the error between the true heading and the estimated heading obtained of the self-localization module.

(11) $$E_{\mathrm{ang}}=\left|\theta_{s}-\theta_{a}\right|$$

where $\theta_{s}$ and $\theta_{a}$ are the self-localized heading and the true heading, respectively. The orientation accuracy of the robot decreases as $E_{\mathrm{ang}}$ increases, and $E_{\mathrm{ang}}=0$ is the ideal case.

### 4.2. Experiment of Effective Self-Localization Area

Restricted by the view field of the visual localization module, the effective area of self-localization that can be provided by the optical beacon to swarm robots is limited. Although the effective localization area can be directly calculated according to the size and height of the optical beacons, the distorted imaging at the edge of the visual field may make beacon verification impossible, leading to a smaller effective localization area than expected in theory. 

**Figure 7:**
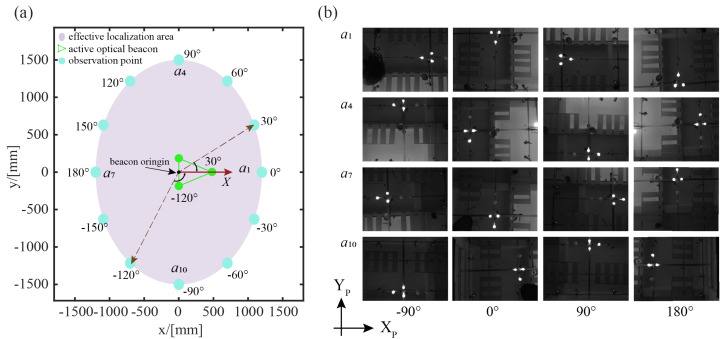
Experimental results of effective self-localization area: (**a**) effective localization area for robots; (**b**) some images observed by robot at different locations.

As seen in Figure 7, we set 12 test directions with 30° intervals and control the robot to move gradually outward from the beacon’s origin point along these directions. As the robot makes each change in position, it slowly rotates in place while observing the beacon in its field of view. The position is considered to be within the effective localization range if the beacon can be fully observed and successfully verified during the robot’s rotation. The experimental results show that the effective localization area is ellipse-like, with a short axis of around 2.4 m and a long axis of around 3.0 m, when the beacon is positioned at a height of 2.7 m. Notably, the effective localization area is roughly 400 times larger than the SwarmBang’s size, meaning that the beacon can support more than 100 robots carrying out tasks simultaneously. Certainly, the effective localization area can be further extended by increasing the height of the beacon.

### 4.3. Experiment of Static Localization

The static localization experiment corresponds to the scenarios in which swarm robots do not need to move their positions during task operation, such tasks as detecting and sampling at certain fixed locations. It is featured that both robots and the beacon are in a stationary state, resulting in a stable and clear imaging result, which facilitates swarm robots to locate their positions and headings with high accuracy.

**Figure 8:**
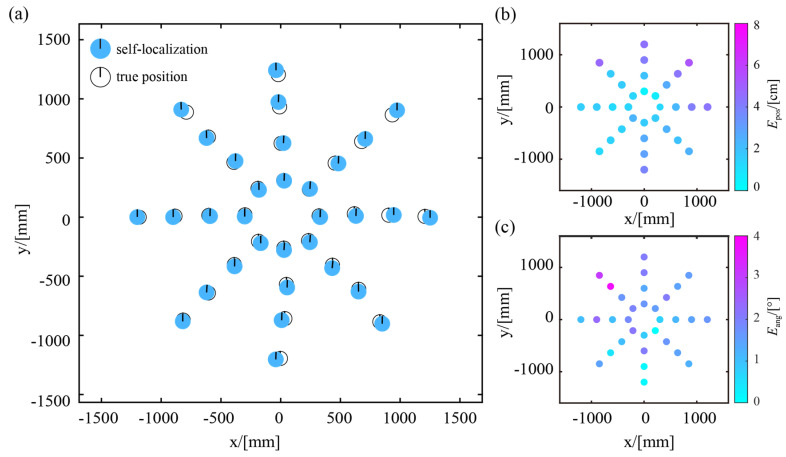
Experimental results of robot without moving: (**a**) true position and self-localization results; (**b**) statistical results of position error; (**c**) statistical results of heading error.

As shown in Figure 8a, the robot is placed at 32 distinct locations within the effective localization range, and these locations are spaced at intervals of 0.3 m, 0.6 m, 0.9 m, and 1.2 m from the origin point. At each location, the robot remains stationary for 30 s, continuously monitoring the beacon and recording the localization results at 0.2 s intervals. The experimental results in Figure 8 are derived from the median values of the observed localization data. On average, the position error is 2.41 cm, and the heading error is 1.44°. Thus, the self-localization method proposed in this paper has a good localization performance when the robot is at rest.

It is worth noting that the positioning accuracy of the robot may become less precise when it is far away from the beacon’s origin. As shown in Figure 8b, when the robot is less than 0.6 m from the origin, the position error ranges from 0.47 cm to 2.69 cm, with an average of 1.68 cm. When the distance from the origin is within 0.6–0.9 m, the position error ranges between 1.50 cm and 4.19 cm, with an average of 2.77 cm. When the robot is located within 0.9–1.2 m from the origin, the position error is between 1.15 cm and 5.39 cm, with an average of 3.5 cm. Once the robot reaches nearly the edge of the effective localization area, the location accuracy may be hindered by camera distortions. This problem can be solved to some extent by adding image distortion correction to the recognition process or by improving the resolution of the camera.

The statistical results of the robot’s orientation are shown in Figure 8c. The distribution of the heading errors ranges primarily from 0.70° to 2.30°, with an average of around 1.50°, indicating that our approach achieves an excellent orientation performance. This is due to the fact that the position error is much smaller than the distance from vertex N to the beacon’s origin, which contributes to reducing the upper limit of heading errors. Therefore, the heading error does not increase dramatically with the robot’s distance from the origin.

### 4.4. Experiment of Dynamic Localization

The dynamic localization experiment corresponds to scenarios in which swarm robots need to move during task operations, such as coordinated formation, collective cleaning, and cooperative transport. The most significant feature, in this case, is the motion blurring of images [30] and the optical axis wobble, which leads to a degradation in the precision of the robot’s localization. To investigate the localization performance of our approach, we first employ a single robot moving along a straight and a square trajectory (each experiment is repeated five times), and then a swarm of 5 robots is employed in the experiment. The robot’s control commands are updated at 0.2 s intervals, and it has a maximum linear speed of 120 mm/s and a maximum angular speed of 42 deg/s.

#### 4.4.1. Straight Trajectory

**Figure 9:**
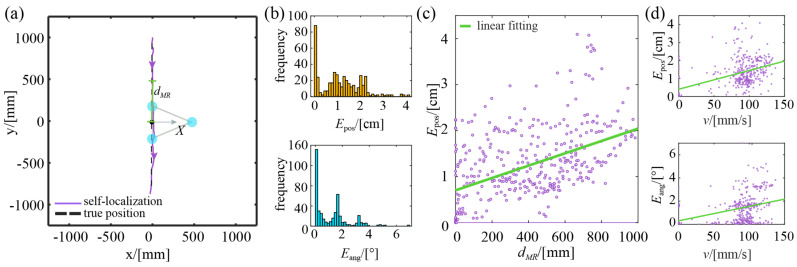
Experimental results of moving robot with the straight trajectory: (**a**) a typical case; (**b**) histograms of position error and heading error; (**c**) spatial distribution of position error; (**d**) the distribution of position error and heading error with different linear speeds.

The experimental results of the robot moving along a straight trajectory are shown in Figure 9, where $d_{MR}$ is the distance from the robot to the origin. It can be seen from the case shown in Figure 9a, that the robot’s self- localization trajectory roughly overlaps with its true trajectory, indicating that good localization results are obtained. According to the quantitative statistics obtained from five repeated trials (see Table 1 for the corresponding data of each trial), as shown in Figure 9b, the robot’s position error is less than 4.09 cm, with an average error of 1.12 cm; the heading error is essentially less than 5.08°, with an average error of 1.16°. Further analysis of the spatial distribution of these errors shows that the robot’s positioning accuracy decreases as its location gets further away from the origin, as shown in Figure 9c. This is caused by the imaging distortion of the visual localization camera.

Figure 9d further shows the relationship between the positioning error, the heading error, and the robot’s linear speed. Clearly, its positioning and heading errors increase as the robot’s linear speed increases. This is mainly due to motion-induced image blur, which increases the beacon recognition errors. This can be reduced to some extent by improving the sampling rate of the camera. 

#### 4.4.2. Square Trajectory

**Figure 10:**
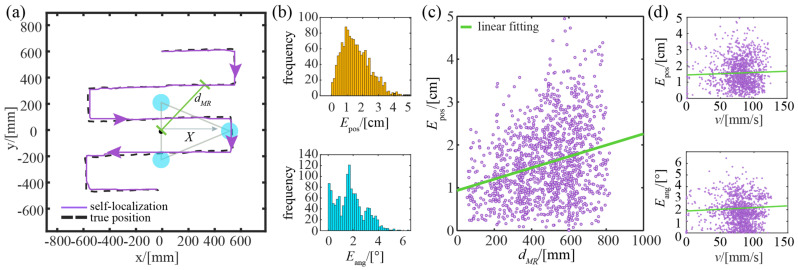
Experimental results of the moving robot with a square trajectory: (**a**) a typical case; (**b**) histograms of position error and heading error; (**c**) spatial distribution of position error; (**d**) the distribution of position error and heading error with different linear speeds.

Figure 10a shows a typical result in which the robot moves along a square trajectory. Generally, the self-localized trajectory matches the true trajectory of the robot, although some noticeable errors are present at certain locations. To get a quantitative picture, this experiment is repeated five times and the corresponding results for each trial are presented in Table 2, while the overall statistics are shown in Figure 10b. Quantitatively, the position error of the robot ranges from 0.01 cm to 4.94 cm, with an average of 1.56 cm; the heading error is less than 6.45°, with an average of 1.79°. Similar to the results in the previous section, Figure 10c shows that the positioning accuracy of the robot decreases gradually as it moves away from the origin. This is mainly due to the imaging distortion at the edges of the camera’s field of view. 

Figure 10d further shows the relationship between the positioning and heading errors and the robot’s linear speed. It can be seen that the robot’s positioning and heading errors slowly increase as the robot’s linear speed increases. This is because the linear speed decreases as the robot turns, at which the optical axis of the camera may drift, causing significant position and heading errors even at low linear speed. We can solve this issue by introducing a horizontal verification process for imaging to reduce the negative effects of the optical axis deviation.

#### 4.4.3. Self-Localization Experiment for a Swarm of Robots

**Figure 11:**
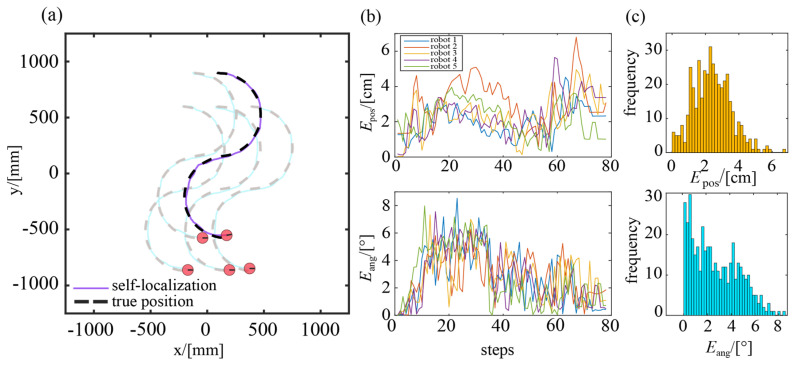
Experimental results of swarm self-localization: (**a**) true position and self-localization results; (**b**) variation curves of position error and heading error; (**c**) histograms of position error and heading error.

In this experiment, a swarm of five robots is employed that moved in a semicircular trajectory. The experimental results are shown in Figure 11. Overall, there is a high degree of coincidence between the self-localized trajectory of robots and their true motion trajectory, indicating a favorable performance of self-localization. Figure 11b depicts the time-evolving curves of the position and headings errors of these robots during their movement. It can be seen that the position errors are less than 6.75 cm, and the heading errors are no greater than 8.53°. Further statistical analysis shows that the average position error is 2.40 cm, with 90% of the position errors ranging from 0.50 cm to 4.20 cm. Meanwhile, the average heading error is 2.66° and 86.7% of the heading errors are less than 5.10°. Table 3 gives the positioning and heading errors for each individual in the robotic swarm. 

Compared to previous experiments in which the robot moves along a straight trajectory, the localization performance of the semicircular trajectory is slightly worse. This is due to the mounting bias of the camera, which causes more significant optical axis shifts when the robot turns its orientation.

## 5. Conclusions

In this paper, a minimalist self-localization approach for miniature swarm robots is proposed by skillfully utilizing the structured ceiling of an indoor environment, particularly taking into account the small size and limited computing power of the swarm robots. A robotic navigator actively projects a triangular optical beacon onto the building’s ceiling to provide localization reference for the robotic swarm. By analyzing the visual information collected by a bottom-up-view camera, the individual robot can determine its position and heading within the beacon coordinate system. Several robotic experiments are conducted to verify the feasibility and effectiveness of our approach. The experimental results show that our approach can provide reliable positioning and heading services for swarm robots with sufficient accuracy to meet the operational needs of swarm robots. 

Our future work will focus on eliminating the negative impacts of the camera mounting bias and the optical distortion on self-localization accuracy. In addition, we will explore the cooperative strategy between swarm robots and robotic navigators to extend the localization range and implement more complex tasks.

## Figures and Tables

**Table 1:** Localization performance of five straight trajectory motion experiments.

No.	Average Position Error (cm)	Position Error Variance	Average Heading Error (°)	Heading Error Variance
1	0.73	0.38	0.83	0.82
2	0.90	0.94	0.74	0.70
3	1.41	0.79	1.04	1.82
4	1.07	0.51	1.41	1.95
5	1.62	0.71	2.03	1.73

**Table 2:** Localization performance of five square trajectory motion experiments.

No.	Average Position Error (cm)	Position Error Variance	Average Heading Error (°)	Heading Error Variance
1	1.41	0.64	1.31	0.72
2	1.49	0.83	2.02	1.12
3	1.81	0.77	1.89	1.34
4	1.53	0.84	1.37	1.08
5	1.55	0.73	2.32	1.48

**Table 3:** Localization performance of swarm self-localization experiments with five robots.

Robot ID	Average Position Error (cm)	Position Error Variance	Average Heading Error (°)	Heading Error Variance
1	2.02	0.66	2.63	4.28
2	3.09	0.42	2.96	2.17
3	2.38	1.24	2.76	3.13
4	2.42	0.60	2.60	4.32
5	2.11	0.89	2.62	4.66

## Data Availability

Not applicable.
